# Association between *Low Density Lipoprotein Receptor-Related Protein 2* Gene Polymorphisms and Bone Mineral Density Variation in Chinese Population

**DOI:** 10.1371/journal.pone.0028874

**Published:** 2011-12-09

**Authors:** Chun Wang, Yi-Ming Hu, Jin-Wei He, Jie-Mei Gu, Hao Zhang, Wei-Wei Hu, Hua Yue, Gao Gao, Wen-Jin Xiao, Jin-Bo Yu, Yao-Hua Ke, Yun-Qiu Hu, Miao Li, Yu-Juan Liu, Wen-Zhen Fu, Ying Ren, Zhen-Lin Zhang

**Affiliations:** 1 Department of Osteoporosis and Bone Diseases, Metabolic Bone Disease and Genetics Research Unit, Shanghai Sixth People's Hospital affiliated with Shanghai Jiaotong University, Shanghai, China; 2 Department of Special Medical Services, Shanghai Sixth People's Hospital affiliated with Shanghai Jiaotong University, Shanghai, China; Maastricht University Medical Center, The Netherlands

## Abstract

*Low density lipoprotein receptor-related protein 2* gene (*LRP2*) is located next to the genomic region showing suggestive linkage with both hip and wrist bone mineral density (BMD) phenotypes. *LRP2* knockout mice showed severe vitamin D deficiency and bone disease, indicating the involvement of *LRP2* in the preservation of vitamin D metabolites and delivery of the precursor to the kidney for the generation of 1α,25(OH)_2_D_3_. In order to investigate the contribution of *LRP2* gene polymorphisms to the variation of BMD in Chinese population, a total of 330 Chinese female-offspring nuclear families with 1088 individuals and 400 Chinese male-offspring nuclear families with 1215 individuals were genotyped at six tagSNPs of the *LRP2* gene (rs2389557, rs2544381, rs7600336, rs10210408, rs2075252 and rs4667591). BMD values at the lumbar spine 1–4 (L1-4) and hip sites were measured by DXA. The association between *LRP2* polymorphisms and BMD phenotypes was assessed by quantitative transmission disequilibrium tests (QTDTs) in female- and male-offspring nuclear families separately. In the female-offspring nuclear families, rs2075252 and haplotype GA of rs4667591 and rs2075252 were identified in the nominally significant total association with peak BMD at L1-4; however, no significant within-family association was found between peak BMD at the L1-4 and hip sites and six tagSNPs or haplotypes. In male-offspring nuclear families, neither the six tagSNPs nor the haplotypes was in total association or within-family association with the peak BMD variation at the L1-4 and hip sites by QTDT analysis. Our findings suggested that the polymorphisms of *LRP2* gene is not a major factor that contributes to the peak BMD variation in Chinese population.

## Introduction

Osteoporosis is a complex disorder with multiple interactions between genetic effects and environmental factors. The heritability of bone mineral density (BMD) is 50–80% [1]. In numerous studies on the genetic mechanisms of osteoporosis, associations between genes involved in vitamin D metabolism and the variation of BMD have been extensively reported [2], [3], [4], [5]. According to genome-wide association studies (GWAS) from the past two years, the highlighted candidate disease-causing genes of osteoporosis, a member of the Wnt signaling pathway, binds to a membrane receptor complex comprising a frizzled (FZD) G-protein-coupled receptor and a low density lipoprotein receptor-related protein (LRP) [6].

The *low density lipoprotein receptor-related protein 2* (*LRP2*) gene, also termed glycoprotein-330 or megalin, is located on chromosome 2q24–q31 and was first reported by Farquhar and colleagues in 1995 [7]. This chromosome region is closely next to the 2q32, the genomic region showing suggestive linkage with both hip and wrist BMD phenotypes[8]. *LRP2* knockout mice showed severe vitamin D deficiency and bone disease [9], indicating the involvement of *LRP2* in the preservation of vitamin D metabolites and delivery of the precursor to the kidney for the generation of 1α,25(OH)_2_D_3_. In 2007, Hsu and colleague [10] firstly suggested that *LRP2* is one of two potential osteoporosis candidate genes in chromosome 2q quantitative trait loci (QTL) region in a Chinese population. There is no evidence, however, that *LRP2* is a member of the LRP family that is involved in the Wnt signaling pathway. The association between *LRP2* and peak BMD variation remains unknown. The aim of this study was to investigate the contribution of *LPR2* gene polymorphisms to peak BMD variation in Chinese female- and male-offspring nuclear families.

## Results

### Basic characteristics of study subjects

Two groups of subjects, 330 female-offspring nuclear families and 400 male-offspring nuclear families, were involved in this study. The detailed basic characteristics of these subjects are summarized in Table 1. The average family size of female- and male-offspring nuclear families were 3.3 (from 3 to 6) and 3.03 (from 3 to 4), respectively.

**Table 1:** Basic clinical characteristics of the parents and adult offspring from 730 nuclear families.

Nuclear families		number	Age (years)	Height (cm)	Weight (kg)	BMI (kg/m^2^)	L1-4 BMD* (g/cm^2^)	Femoral neck BMD* (g/cm^2^)	Total hip BMD* (g/cm^2^)
female-offspring nuclear families	Father	330	63.2±7.6	166.8±6.1	70.0±10.1	25.1±3.2	1.139±0.182	0.886±0.122	0.958±0.126
	Mother	330	60.5±7.1	155.4±5.6	59.3±9.0	24.6±3.5	0.985±0.166	0.790±0.126	0.849±0.13
	Daughter	428	34.5±6.9	159.8±5.1	55.6±7.7	21.8±2.9	1.174±0.134	0.936±0.114	0.969±0.116
male-offspring nuclear families	Father	400	61.1±7.1	167.8±6.0	69.7±9.5	25.2±2.7	1.139±0.171	0.892±0.132	0.958±0.138
	Mother	400	58.4±6.3	155.7±5.5	58.2±8.2	24.0±3.1	0.992±0.168	0.796±0.144	0.852±0.162
	Son	415	30.4±6.1	172.9±5.9	70.7±10.8	24.2±3.2	1.138±0.137	0.995±0.141	1.008±0.142

*The reported BMD values (mean±SD) are unadjusted raw BMD values.

### Genotype frequencies and linkage disequilibrium

All subjects were genotyped at six chosen tagSNPs. The MAF of each tagSNP was agreed with the HapMap data of Chinese population. The distribution of each genotype is in Hardy-Weinberg equilibrium.

**Figure 1:**
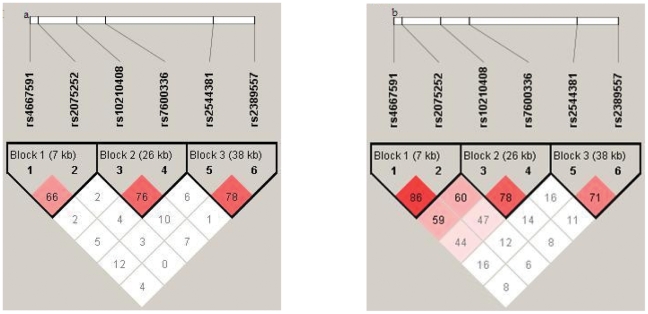
Haplotype linkage disequilibrium (LD) blocks in 330 female-offspring nuclear families and 400 male-offspring nuclear families. Three LD blocks connecting SNP pairs are shaded based on the LD strength between SNPs by using the disequilibrium coefficient r^2^, darker shades of red indicate higher value of D', up to maximum of 1. Figure 1a shows the LD pattern in the *LRP2* gene based on the parents genotypes from 330 female-offspring nuclear families (n = 660), while figure 1b presents the LD pattern from 400 male-offspring nuclear families (*n* = 800).

Three haplotype blocks were defined according to the LD value in both female- and male-offspring nuclear families. In female-offspring nuclear families, the LD pattern of block 1–3 are shown in Figure 1a. The highest LD value of 0.78 was observed between rs2544381 and rs2389557. In male-offspring nuclear families, the LD pattern of block 1–3 are shown in Figure 1b. The highest LD value of 0.86 was observed between rs4667591 and rs2075252.

### Peak BMD phenotypes and genotypes in 330 unrelated adult daughters and 400 unrelated adult sons from the studied nuclear families

The peak BMD values at the L1-4 and hip sites and the genotypes of the six tagSNPs are listed in Table 2. The reported BMD values (mean±SD) were previously adjusted by age, height and weight as covariates. There was no significant association between genotypes and peak BMD phenotypes using GLM-ANOVA.

**Table 2:** Peak BMD phenotypes and genotypes in 330 unrelated daughters and 400 unrelated adult sons from the studied nuclear families.

SNP	genotype	female-offspring nuclear families	n	L1-4(g/cm^2^)	Femoral neck (g/cm^2^)	Total hip (g/cm^2^)	male-offspring nuclear families	n	L1-4(g/cm^2^)	Femoral neck (g/cm^2^)	Total hip (g/cm^2^)
rs2389557	TT		92	1.183±0.012	0.944±0.010	0.972±0.010		120	1.139±0.012	0.998±0.012	1.017±0.012
	CC		66	1.186±0.014	0.931±0.012	0.968±0.012		94	1.169±0.013	1.018±0.013	1.030±0.013
	CT		172	1.164±0.009	0.934±0.007	0.968±0.007		186	1.126±0.010	0.987±0.009	1.006±0.009
rs2544381	GG		186	1.173±0.008	0.935±0.007	0.965±0.007		226	1.135±0.009	0.993±0.008	1.010±0.008
	CC		14	1.212±0.031	0.932±0.026	0.98±0.026		26	1.133±0.026	0.993±0.025	1.014±0.024
	GC		130	1.170±0.010	0.939±0.008	0.975±0.008		148	1.150±0.011	1.006±0.011	1.023±0.010
rs7600336	CC		88	1.171±0.012	0.929±0.010	0.968±0.010		118	1.140±0.012	0.994±0.012	1.103±0.012
	TT		55	1.187±0.016	0.929±0.013	0.965±0.013		72	1.142±0.016	1.000±0.015	1.019±0.015
	CT		187	1.171±0.008	0.942±0.007	0.972±0.007		210	1.140±0.009	0.999±0.009	1.015±0.009
rs10210408	TT		55	1.175±0.015	0.941±0.013	0.976±0.013		56	1.157±0.018	0.994±0.017	1.012±0.017
	CC		116	1.185±0.011	0.936±0.009	0.972±0.009		133	1.142±0.011	0.998±0.011	1.019±0.011
	CT		159	1.165±0.009	0.935±0.008	0.965±0.008		211	1.135±0.009	0.998±0.009	1.014±0.009
rs2075252	AA		90	1.188±0.012	0.937±0.010	0.974±0.010		135	1.144±0.011	0.999±0.011	1.019±0.011
	GG		63	1.146±0.014	0.933±0.012	0.956±0.012		65	1.154±0.016	1.014±0.016	1.025±0.015
	AG		177	1.176±0.009	0.937±0.007	0.971±0.007		200	1.134±0.009	0.992±0.009	1.010±0.009
rs4667591	TT		101	1.163±0.012	0.931±0.010	0.959±0.010		157	1.141±0.011	0.999±0.010	1.019±0.010
	GG		67	1.188±0.014	0.937±0.011	0.976±0.011		54	1.151±0.019	0.991±0.018	0.994±0.018
	TG		162	1.174±0.009	0.939±0.007	0.973±0.007		189	1.137±0.010	0.997±0.009	1.013±0.009

### Association between SNPs and haplotypes with peak BMD variation in female- and male-offspring nuclear families

The association between peak BMD and six tagSNPs or three haplotype blocks in the *LRP2* gene using QTDT analysis are summarized in Table 3 and 4. There were 271, 236, 266, 271, 263 and 253 informative female-offspring nuclear families and 307, 238, 310, 310, 279 and 238 informative male-offspring nuclear families for the QTDT analysis at rs2389557, rs2544381, rs7600336, rs10210408, rs2075252 and rs4667591, respectively. No population stratification was found for the six tagSNPs and haplotypes in the study subjects.

**Table 3:** *P* values obtained for analyses of population stratification, total association and within-family association for six tagSNPs.

	rs2389557	rs2544381	rs7600336	rs10210408	rs2075252	rs4667591
Test of population stratification in female-offspring nuclear families						
L1-4	0.768	0.458	0.112	0.354	0.796	0.416
Femoral neck	0.327	0.931	0.648	0.550	0.898	0.545
Total hip	0.982	0.258	0.685	0.379	0.637	0.293
Test of total association in female-offspring nuclear families						
L1-4	0.333	0.086	0.188	0.575	0.040*	0.154
Femoral neck	0.228	0.363	0.868	0.987	0.690	0.186
Total hip	0.462	0.796	0.907	0.936	0.172	0.099
Test of within-family association in female-offspring nuclear families						
L1-4	0.390	0.103	0.720	0.286	0.136	0.083
Femoral neck	0.993	0.544	0.645	0.652	0.890	0.217
Total hip	0.623	0.291	0.700	0.539	0.631	0.070
Test of population stratification in male-offspring nuclear families						
L1-4	0.491	0.860	0.127	0.275	0.351	0.408
Femoral neck	0.277	0.765	0.659	0.332	0.545	0.235
Total hip	0.292	0.433	0.887	0.837	0.915	0.753
Test of total association in male-offspring nuclear families						
L1-4	0.593	0.649	0.796	0.804	0.737	0.647
Femoral neck	0.878	0.892	0.751	0.578	0.933	0.904
Total hip	0.603	0.914	0.385	0.267	0.603	0.456
Test of within-family association in male-offspring nuclear families						
L1-4	0.753	0.949	0.146	0.407	0.492	0.566
Femoral neck	0.387	0.741	0.589	0.264	0.610	0.296
Total hip	0.526	0.461	0.578	0.715	0.748	0.558

*p<0.05.

**Table 4:** *P* values obtained for analyses of population stratification, total association and within-family association for four haplotypes of block 1–3.

	Block 1					Block 2					Block 3			
	TA	GG	TG	GA		CC	TT	CT	TC		GT	GC	CC	CT
Test of population stratification in female-offspring nuclear families														
L1-4	0.451	0.117	0.972	0.603		0.144	0.717	0.348	0.303		0.204	0.295	0.343	0.827
Femoral neck	0.342	0.382	0.202	0.956		0.672	0.634	0.846	0.106		0.303	0.261	0.575	0.103
Total hip	0.745	0.113	0.393	0.521		0.615	0.413	0.653	0.301		0.525	0.520	0.647	0.409
Test of total association in female-offspring nuclear families														
L1-4	0.423	0.524	0.262	0.021*		0.812	0.078	0.555	0.445		0.357	0.156	0.072	0.443
Femoral neck	0.389	0.434	0.987	0.194		0.945	0.896	0.942	0.607		0.406	0.603	0.651	0.325
Total hip	0.682	0.375	0.921	0.503		0.678	0.231	0.833	0.819		0.271	0.945	0.808	0.839
Test of within-family association in female-offspring nuclear families														
L1-4	0.272	0.403	0.442	0.074		0.212	0.343	0.272	0.766		0.338	0.122	0.266	0.401
Femoral neck	0.893	0.844	0.363	0.483		0.713	0.806	0.845	0.344		0.479	0.753	0.338	0.405
Total hip	0.602	0.473	0.601	0.365		0.925	0.841	0.823	0.521		0.692	0.519	0.512	0.692
Test of population stratification in male-offspring nuclear families														
L1-4	0.302	0.349	0.763	1.000		0.213	0.087	0.958	0.334		0.321	0.393	0.674	0.789
Femoral neck	0.524	0.453	0.845	0.832		0.364	0.473	0.948	0.405		0.325	0.389	0.435	0.299
Total hip	0.967	0.889	0.512	0.869		0.507	0.779	0.247	0.389		0.348	0.821	0.101	0.513
Test of total association in male-offspring nuclear families														
L1-4	0.724	0.981	0.553	0.799		0.745	0.937	0.593	0.839		0.968	0.976	0.988	0.980
Femoral neck	0.810	0.814	0.499	0.621		0.544	0.703	0.989	0.558		0.687	0.958	0.679	0.986
Total hip	0.583	0.393	0.507	0.994		0.323	0.238	0.724	0.891		0.810	0.613	0.570	0.501
Test of within-family association in male-offspring nuclear families														
L1-4	0.445	0.393	0.964	0.979		0.213	0.151	0.759	0.434		0.392	0.446	0.714	0.821
Femoral neck	0.633	0.426	0.618	0.990		0.266	0.409	0.960	0.324		0.301	0.432	0.379	0.344
Total hip	0.781	0.817	0.815	0.892		0.292	0.728	0.413	0.405		0.348	0.659	0.082	0.753

*p<0.05.

In female-offspring nuclear families, rs2075252 and haplotype GA of rs4667591 and rs2075252 were identified in nominally significant total association with peak BMD at L1-4 (*p* = 0.040 and 0.021, respectively). However, neither SNPs nor haplotypes was found in the significant within-family association with peak BMD at the L1-4 and hip sites in the same group.

In male-offspring nuclear families, for the total and within-family associations, six tagSNPs were associated with neither the variation of the L1-4 BMD nor the hip BMD (femoral neck and total hip). Haplotype analysis revealed similar negative results. No significant total association or within-family association was found between any of the haplotypes in three blocks and the variations of peak BMD at the L1-4 and hip sites.

## Discussion

Although osteoporosis mainly occurs in the senior population, this disease is largely determined by the peak bone mass that is achieved in adulthood. In Chinese men and women, peak BMD at the lumbar spine and hip sites is attained between the ages of 20 and 39 years [11], [12]. Peak BMD is strongly controlled by genetic determinants. The heritability estimates in our male-offspring, calculated by parent-offspring regression [2], [11], for peak BMD at the lumbar spine, femoral neck and total hip were 0.565, 0.702 and 0.693, respectively. In our female-offspring, the heritability estimates for peak BMD at the lumbar spine, femoral neck and total hip were 0.545, 0.504 and 0.501, respectively.

Many association studies have been performed to discover the genetic determinants of BMD. Estrogen receptor 1(ESR1), ZBTB40, Tumor necrosis factor receptor superfamily 11B (TNFRSF11B), Low density lipoprotein receptor-related protein 5 (LRP5), SP7, Tumor necrosis factor superfamily 11(TNFSF11) and Tumor necrosis factor superfamily 11A (TNFSF11A) are seven known loci associated with BMD at the GWAS level. The knowledge with respect to the contribution of these seven loci to the variation of BMD, however, is still limited [13], [14], [15]. To date, the major genetic factors that influence BMD are unknown. Many studies have suggested that BMD may influenced by numerous minor genetic markers [16], [17]. 1α,25(OH)_2_D_3_, which is the active form of vitamin D, plays an important role in bone metabolism, including bone mineralization, modeling and remodeling, by regulating osteoblast and osteoclast functions [18], [19]. A deficiency of 1α,25(OH)_2_D_3_ may result in osteoporosis, fragility fractures and other health problems [20], [21]. Currently, extensive studies on the vitamin D receptor gene (VDR) as an osteoporosis candidate gene have yielded many contradictory results [2], [22], [23], [24], [25], [26], [27], [28]. Recently, Lou and colleagues [29] reported that 25OHD_3_ acts as an agonist of the vitamin D receptor ligand and has a synergistic effect with 1α,25(OH)_2_D_3_ and that high expression of the LRP2 gene was found in Cyp27b1 gene knockout cells. Because the Cyp27b1 gene encodes 1α-hydroxylase, which is necessary for the activation of 25OHD_3_, this group suggested that 25OHD_3_ is not only a prehormone of 1α,25(OH)_2_D_3_ but also plays a role in cell proliferation and gene regulation.

The *LRP2* gene is known as a virulence gene in Heymann nephritis [30]. In 1999, Nykjaer and colleagues [9] first reported that compounds of 25OHD_3_ and the vitamin D-binding protein (DBP) were filtered through the glomerulus and reabsorbed by *LRP2* into the proximal tubular cells in animal studies. In *LRP2* knockout mice, vitamin D deficiency and bone disease were induced by the abnormal urinary excretion of 25OHD_3_ and DBP. Therefore, a renal uptake pathway that is essential for the preservation of vitamin D metabolites and the delivery of the precursor for the generation of 1α,25(OH)_2_D_3_ was defined. The above-mentioned studies suggested that the *LRP2* gene is involved in the achievement and maintenance of peak BMD through the function of 25OHD_3_.

To the best of our knowledge, this is the first study to investigate the association between *LRP2* gene polymorphisms and peak BMD variation in female- and male-offspring nuclear families. Six tagSNPs of the *LRP2* gene were identified in 330 female-offspring nuclear families with 1088 individuals and 400 male-offspring nuclear families with 1215 individuals. The distribution frequencies of these SNPs were in agreement with data from HapMap Asian populations. BMD values at the L1-4 and hip sites were measured individually as the phenotypes.

In female-offspring nuclear families, rs2075252 and haplotype GA of rs4667591 and rs2075252 were identified in nominally significant total association with peak BMD at lumbar spine. Unfortunately, no within-family association between peak BMD variation and SNPs or haplotypes was found in the same group. In male-offspring nuclear families, neither the six tagSNPs nor the haplotypes was in total association or within-family association with the peak BMD variation at the L1-4 and hip sites by QTDT analysis. The statistical power was estimated by the Piface program (version 1.65 http://www.math.uiowa.edu/~rlenth/Power/) at the time that this study was designed. In our previous association studies of 401 female-offspring nuclear families with 1260 subjects and candidate genes (*Estrogen receptor alpha* and *Myostatin*), the sample size offered more than 80% power and explained about 10% of the BMD variation [31], [32]. Based on the MAF of each SNP in this study, 330 female-offspring nuclear families with 1088 individuals and 400 male-offspring nuclear families with 1215 subjects had sufficient power to detect any association between the peak BMD and SNPs or haplotype blocks and in Chinese population. Furthermore, five of the studied tagSNPs had a MAF that was very close to or higher than 40%. With greater heterozygosity, more information can be derived from families by QTDT analysis.

To avoid false-positive results, two kinds of statistical analysis were employed in the current study. QTDT analysis examines the transmission of alleles from parents to offspring. Unlike ANOVA analysis, which can be affected by population stratification, the QTDT analysis eliminates this stratification. The within-family association test of QTDT can also eliminate the effect of an admixed and stratified population. Therefore, this analysis is more powerful than ANOVA analysis. Moreover, haplotype analysis is generally thought to be more powerful than analyses that employ single markers. The total and within-family associations were also tested between three haplotype blocks and BMD by QTDT in the nuclear families. Thus, the results of this study should be reliable.

In summary, neither the six tagSNP polymorphisms nor the three haplotype blocks of the *LRP2* gene were associated with peak BMD variation in either male or female Chinese population. This negative result indicates that *LRP2* is not the major contributor of osteoporosis.

## Materials and Methods

### Study subjects

This study was approved by the Ethics Committee of Shanghai Sixth People's Hospital, affiliated with Shanghai Jiaotong University. All subjects involved in the study were recruited by the Department of Osteoporosis and Bone Diseases from a community center in Shanghai from 2004 to 2010. Two groups of subjects were recruited, and the inclusive and exclusive criteria were described previously [2], [11]. Briefly, 330 female-offspring nuclear families with 1088 individuals and 400 male-offspring nuclear families with 1215 individuals were included in the study. Each nuclear family has at least one healthy daughter or son aged from 20 to 40 years old. All recruited daughters were premenopausal. The exclusion criteria for the study subjects were a history of: (1) serious residual effects of cerebral vascular disease; (2) diabetes mellitus except for easily controlled type 2 diabetes mellitus (defined as adult asymptomatic hyperglycemia controlled by diet or oral agents); (3) chronic renal disease (serum creatinine ≥11.9 mg/dl); (4) chronic liver disease; (5) chronic lung disease; (6) corticosteroid therapy at pharmacologic levels for at least 12 weeks; (7) anticonvulsant therapy for at least 16 months; (8) evidence of other metabolic or inherited bone diseases (eg, hyper- or hypoparathyroidism, Paget's disease, osteomalacia or osteogenesis imperfecta); (9) rheumatoid arthritis or collagen disease; (10) major gastrointestinal disease (eg, peptic ulcer, malabsorption, chronic ulcerative colitis, regional enteritis, or any signifcant chronic diarrhea state); (11) signifcant disease of any endocrine organ that would affect bone mass (eg, hyperthyroidism, etc); (12) any neurologic or musculoskeletal condition that would be a nongenetic cause of low bone mass; (13) menopause before 40 years old and (14) any disease, treatment or condition that would be a nongenetic cause of low bone mass. After signing an informed consent form, information on medical history, family history, physical activity, developmental history, childbearing history, alcohol use, smoking history and dietary habits including calcium intake were collected by questionnaire.

### BMD measurement

BMD of the anteroposterior lumbar spine 1–4 (L1-4) and left proximal femur, including the femoral neck and total hip, were measured using a lunar prodigy dual energy X-ray absorptiometry (DXA) densitometer (GE Healthcare, Madison, WI, USA). The data were analyzed using Prodigy enCORE software (ver. 6.70, standard-array mode; GE Healthcare, Madison, WI, USA). The machine was calibrated daily, and the coefficient of variability (CV) values of the DXA measurements (obtained from triplicate measurements of the same 15 individuals) at L1-4, total hip, femoral neck and trochanter were 1.39%, 0.70%, 2.22% and 1.41%, respectively [33]. The long-term reproducibility of our DXA data during the trial based on weekly repeated phantom measurements was 99.55%[32]. Height and body weight were measured using standardized equipment. Body mass Index (BMI) was defined as weight/height^2^ in kg/m^2^.

### SNP selection and genotyping

The selection of evaluated tagSNPs depended on the HapMap database. In order to draw a reliable conclusion, the recruitment criteria were essential: (1) minor allele frequency (MAF) >0.10; (2) r^2^ = 0.8; (3) tagSNPs in the exon region, exon-intron splice conjunction, 5′ flanking region or 3′ untranslated region (UTR). Hence, the only two tagSNPs in the exon region (rs4667591 in exon 69 and rs2075252 in exon 66) and four tagSNPs in the intron region (rs2389557, rs2544381, rs7600336 and rs10210408) spanning the *LRP2* gene were selected for the following association study.

Genomic DNA was isolated from peripheral blood leukocytes using the conventional phenol-chloroform extraction method. Genotypes were identified by the TaqMan assay (Applied Biosystems, Foster City, CA, USA) using the Applied Biosystems SNP assay-by-design service according to the manufacturer's instructions. One allelic probe was labeled with fluorescent FAM dye and the other with fluorescent VIC dye.

### Haplotype and LD analysis

Haplotypes were constructed from the population genotypic data by the algorithm of Stephens using Phase program version 2.0.2. The significance level for LD between the *LRP2* gene markers was assessed according to the observed haplotype and allelic frequencies using Haploview version 3.2. The Lewontin's D' and LD coefficient *r* ^2^ between all pairs of biallelic loci were examined.

### Statistical analyses

Genotype frequencies were tested against Hardy-Weinberg equilibrium by the χ^2^ test to estimate the population homogeneity of the study subjects. To ensure unrelated individual samples, only genotype data from the 1460 parents of the nuclear families were used in the statistical analysis [31].

The quantitative transmission disequilibrium test (QTDT) program, available at `http://www.sph.umich.edu/csg/abecasis/QTDT/, was used to determine population stratification, total family association and within-family association between SNPs and haplotypes and the BMD phenotypes in female- and male-offspring families separately. This method [32], [34], [35] extends the trio-based TDT to quantitative trait data and uses genotypic data from available sibling and parents. Sex was not used as a covariate during analysis because only healthy sons or daughters were recruited in the study families and the effects of the parental phenotypes were already excluded.

One-way ANOVA and general linear model-ANOVA (GLM-ANOVA) were also employed to test the association between genotypes and peak BMD in 330 unrelated adult daughters and 400 unrelated adult sons from the studied nuclear families. Statistical analyses were performed using the SPSS package, version 17.0 (SPSS, Chicago, IL, USA).

In all above-mentioned statistical analyses, BMD values were previously adjusted by age, height and weight as covariates. *P*<0.05 was considered significant for all analyses.
